# Factors for Satisfaction Among Providers of Ancillary Health Services in a Community-Based Cancer Prevention Program: A Pilot Study in Nevada

**DOI:** 10.1100/tsw.2006.151

**Published:** 2006-06-30

**Authors:** Emmanuel C. Gorospe, Christopher R. Cochran, Elena E. Cabb

**Affiliations:** Department of Health Care Administration and Policy, School of Public Health, University of Nevada Las Vegas, USA

**Keywords:** outpatient health services, cancer prevention, indigent care, delivery of health care, United States

## Abstract

Providers of ancillary health services are essential members of any health care delivery system. They supply laboratory, radiology, and other diagnostic modalities necessary for quality medical care. Assessment of the providers' factors for satisfaction in participating in cancer prevention programs can contribute to better services and can serve as a model for other community-based health programs.We conducted a pilot survey of providers of ancillary services in the Nevada Women's Health Connection, a community breast and cervical cancer prevention program. Of the 93 participating providers, a total of 44 providers completed the survey. We subjected the survey data to factor analysis using iterative principal axis factoring with Varimax rotation. Three components of satisfaction were identified, comprising satisfaction with the (1) reimbursement process, (2) positive perception of the program, and (3) familiarity with program's requirements. All three components accounted for 72.08% of the total variance before the rotation. Amount of financial gain was not a significant factor for satisfaction among participating providers. Providers of ancillary health services were satisfied in their participation in this community-based cancer prevention program. There were three components of satisfaction identified. Further attention should be given on these issues as they have implications for quality improvement in health services for community-based programs dealing with low income and uninsured patients.

## INTRODUCTION

Providers of ancillary health services are essential components in any health care delivery system. They render laboratory, radiology, and other diagnostic modalities that are vital to the work of primary care physicians. Previous studies have investigated the measures of satisfaction among physicians[1,2], but have failed to look at the important needs of those who provide ancillary health services. For programs serving low-income and uninsured populations, assessment of provider satisfaction is an important strategy for quality improvement[3,4].

We evaluated the factors for satisfaction among providers of ancillary health services in the Nevada Women's Health Connection (WHC) program, a state-wide, community-based breast and cervical cancer screening for uninsured and low-income women. The WHC program is funded through a grant from the U.S. Centers for Disease Control and Prevention (CDC) as part of the National Breast and Cervical Cancer Early Detection Program (NBCCEDP). The goal of the NBCCEDP is to increase access to breast and cervical cancer screening among women in the U.S.[5]. NBCCEDP supports the national agenda of Healthy People 2010, which aims to eliminate racial health disparities among minorities, the uninsured, and the medically underserved in the U.S.[6,7].

In the WHC program, providers of ancillary services support the cancer screening activities of primary care physicians by administering and relaying results of diagnostics tests. These services are reimbursed by the WHC program. The majority of these ancillary services include mammography, Pap smear cytology, and breast and cervical biopsies. The importance of these services has been highlighted in a related survey that indicated that physicians participating in cancer prevention programs were more satisfied if they were able to receive timely and accurate diagnostic results[8].

Since 1990, the state of Nevada has experienced an enormous population growth with a cumulative growth rate of 81%, primarily through interstate and international migration[9]. Most of the population growth occurs in the Las Vegas metropolitan area due to the attraction of the local hotel, entertainment, and gaming industries. This growth has caused Nevada to lag behind in many health care indicators. Nevada, having one of the largest population of uninsured adults, also has one of the highest tobacco and alcohol consumption rates and other unhealthy lifestyles in the U.S.[10]. In terms of health manpower, Nevada ranks 48$^{th}$ among the 50 U.S. states in the number of physicians per population[11].

Breast cancer ranks as the primary cause of cancer morbidity among women in the U.S.[12]. Early breast and cervical cancer detection prevents excess morbidity and mortality cancer rates, eliminates unnecessary invasive procedures, and reduces health care expenditures. Nevada ranks 15$^{th}$ in the nation for cancer mortality rates[13]. Coinciding with Nevada's population growth, the National Cancer Institute (NCI) predicts that a significant number of women in the state may be expected to develop breast and cervical cancer in the coming years[14]. The prevalence of unhealthy lifestyle behaviors, increasing number of insured constituents, and limited number of health care providers justify the need to identify areas for improvement in the WHC to be effective in its services.

By understanding the needs of health care providers, strategies may be implemented to enhance their satisfaction and guarantee continued support for the program[10,15]. The continued participation of health care providers can also be enhanced by targeting issues that are identified to affect their satisfaction directly in participating with community-based health programs[4,16]. In the private health care industry, satisfied providers in managed care networks are influential in retaining and recruiting highly needed health personnel[17]. Community-based programs must be able to recruit and retain satisfied health providers in mainstream clinical and ancillary health services. Continued support from providers of ancillary health services is crucial in a state like Nevada where medical services and manpower for health are limited.

## METHODS

### Instrument

A needs-and-satisfaction questionnaire was developed based on information obtained from WHC program staff, CDC performance measures, other breast and cervical cancer survey instruments, and literature review. A cover letter was included explaining the need to obtain the providers' needs and satisfaction assessment in order to improve the quality of services for WHC patients. Providers of ancillary health services were asked to complete the questionnaire, assisted by a small paragraph of instruction at the beginning of the page. They were asked to rate their level of agreement to the questions asked on a 5-point Likert scale starting with 1 as “strongly disagree” going up to 5 as “strongly agree”. We inquired about their demographic data, the type of services they offer, their relationship with the WHC program such as communication with the program staff, provision of training opportunities, administrative requirements, and reimbursement issues.

### Participants

A list of laboratory, radiology, and surgical facilities and their administrative directors was obtained from the Nevada State Health Division. To assure adequate experience and familiarity with the program, we only included providers of ancillary services who had participated in the program at some point between the years 1997–2003. We identified a total of 93 potential respondents throughout Nevada.

### Survey

The ethics review and approval to carry out this study was granted by the Nevada State Health Division. The questionnaires were first mailed in October 2004, which yielded 21 responses. Follow-up surveys were faxed to providers who had not responded to the first mailing. The survey lasted over a period of 2 months and a total of 44 completed surveys were collected.

### Statistical Analysis

Survey data were analyzed using SPSS® version 13 statistical analysis software. Frequency rates, means, and standard deviations were obtained on relevant questions pertaining to factors for satisfaction. There were ten questions, probing possible factors for satisfaction. We subjected the survey data to factor analysis in order to uncover latent variables and to merge the different responses to produce the principal components that could explain the observed variance in the survey. Components were extracted using iterative principal axis factoring. The Kaiser-Guttman and Scree tests were conducted to determine the number of components. These tests yielded three components and were subjected to Varimax rotation. Only factors with highly significant loading (>0.7) were included in the interpretation of component contents.

## RESULTS

Forty-nine per cent of the respondents were from Southern Nevada, which includes the Las Vegas metropolitan area. Northern Nevada accounted only for 12% of responses. The rest represented the different rural Nevada counties in small fractions. This distribution represents the concentration of Nevada’s population and health care resources, since the majority of Nevada’s population resides in Las Vegas, the fastest growing metropolis in the state. Among the providers surveyed, 33% provided multiple diagnostic services. The rest were either facilities for mammography, laboratories, and centers where cervical or breast biopsies are done.

We performed a factor analysis on the questions related to the provider satisfaction with the program. With 44 respondents, the Kaiser-Meyer-Olin measure of sampling adequacy was 0.670, suggesting satisfaction with our sample for factor analysis. Bartlett’s test of sphericity was significant at $p < 0.05$. Each item’s mean and standard deviation may be found in Table 1.

**TABLE 1**
**Means and Standard Deviation of Items for Provider Satisfaction**

| Items | Mean | Std. Deviation |
|---|---|---|
| Comparable reimbursement to other payers | 2.77 | 0.961 |
| Similar reimbursement procedures compared to other payers | 3.02 | 1.023 |
| Staff satisfaction to reimbursement process | 3.05 | 1.011 |
| Staff general satisfaction with program | 3.95 | 0.963 |
| Perception of program's benefit to patients | 4.43 | 0.925 |
| Satisfaction with the program's communication | 3.52 | 1.089 |
| Familiarity with program's reporting requirements | 3.93 | 1.149 |
| Familiarity with program services | 3.91 | 1.007 |
| Ease of documentation completion | 4.05 | 0.963 |
| Reimbursement representing a significant portion of the provider's operating budget | 2.05 | 1.160 |

n = 44 respondents.

On a 1–5 Likert scale, which ranges from "dissatisfied" to "strongly satisfied", the highest mean was 4.43 on the item regarding the providers' perception of the WHC program's benefit to their patients. Overall, the provider satisfaction grand mean was 34.68. In this survey, the highest possible score for provider satisfaction was 50.

The Kaiser-Guttman and Scree tests indicated that three components from the 10-item questionnaire best fitted these data. All three components accounted for 72.08% of the total variance before the rotation. The three components or dimensions in order of importance were satisfaction with the (1) reimbursement process, (2) positive perception of the program, and (3) familiarity with the program's requirements (see Table 2).

**TABLE 2**
**Provider Satisfaction Pattern Matrix**

| | Reimbursement Process | Positive Perception | Familiarity with Program Requirements |
|---|---|---|---|
| Comparable reimbursement to other payers | 0.876 | * | * |
| Similar reimbursement procedures compared to other payers | 0.841 | * | * |
| Staff satisfaction to reimbursement process | 0.713 | * | * |
| Staff general satisfaction with program | * | 0.875 | * |
| Perception of program's benefit to patients | * | 0.722 | * |
| Satisfaction with the program's communication | * | 0.706 | * |
| Familiarity with program's reporting requirements | * | * | 0.896 |
| Familiarity with program's services | * | * | 0.818 |
| Ease of documentation completion | * | * | 0.725 |
| Reimbursement representing a significant portion of the provider's operating budget | * | * | * |
| **Eigenvalues** | **3.50** | **2.53** | **1.18** |

* Factors with <0.7 loading have been suppressed; extraction method: Principal Component Analysis; rotation method: Varimax with Kaiser Normalization; n = 44 respondents.

Three items were significantly loaded on the first component regarding the reimbursement process. These included: (1) comparable reimbursement to other payers, (2) similar reimbursement procedures compared to other payers, and (3) staff satisfaction to reimbursement process. There were also three items loaded on the second component which pertains to the (1) staff satisfaction with the program, (2) perception of the program's benefit to patients, and (3) satisfactory communication with the program staff. The third satisfaction component consists of (1) familiarity with reporting requirements, (2) familiarity with the program's services, and (3) ease of documentation completion. Financial gain in such a way that the program's reimbursement represented a significant portion of the provider's operating budget did not load significantly in any of the components identified.

## DISCUSSION

In this survey, we identified three components or dimensions for satisfaction among providers of ancillary health services. The satisfaction dimension most important to the providers was the process of reimbursement. This measure of satisfaction directly refers to the procedure of availing reimbursement and not to the actual amount of compensation.

For community-based health programs such as the WHC, an important implication is that ancillary service providers will be more satisfied with their participation if they are content with the process of how they are reimbursed regardless if the reimbursement comprises a significant source of revenue in their practice. It is possible that health care providers may have lesser expectations for financial gain, especially from government programs intended for the care of indigent and uninsured patients[18]. Given the current trend for decreasing reimbursements in both private and public health insurance systems, health care providers probably expect more efficient reimbursement procedures to compensate for their less-than-expected revenue from service-oriented community health programs. Unnecessary delays and cumbersome procedures for reimbursement create extra burden to the administrative staff, which may require reorganization of staff duties, hiring of additional employees, and bigger operational expenses[19].

The second dimension for provider satisfaction was related to having a positive general perception about the WHC program. Having a positive outlook may enhance participating providers' morale and sense of job fulfillment. Well-motivated health care providers are indispensable for a well-functioning health care organization[20]. Beyond financial issues, a community-based health program must be perceived as beneficial to health care providers as well as socially relevant to their indigent clientele. In addition to program perception, satisfaction with the communication with program staff was also an essential component. Good communication would facilitate better coordination of care and resolution of any management issues. Even in a profit-oriented managed care setting, satisfactory communication fosters a sense of support and cooperation among the providers and managed care staff[19].

Lastly, familiarity with program's services and documentation requirements constitute an important contribution to provider satisfaction. Awareness of the program's services allows providers a better understanding of what health care benefits they can offer to their low-income patients without any restriction from lack of insurance coverage. Together with the understanding of the program's services comes the need to have efficient and simple documentation procedures. Our results support studies that suggest that increased documentation negatively affects health care provider satisfaction[19,21] and willingness to participate in similar programs for patients with limited health care resources[18].

## CONCLUSION

Providers of ancillary services were satisfied with their participation in this community-based cancer prevention program. To further enhance their satisfaction, attention must be given to the principal factors for provider satisfaction. Based on their responses, these factors include satisfaction with the reimbursement process, positive program perception, and familiarity with program requirements.

One of the limitations of this study is that it only examined ancillary service providers in Nevada's breast and cervical cancer screening program, who may not be representative of the NBCCEDP ancillary service providers in general. However, current research on satisfaction of ancillary health service providers is generally limited. Thus, this study may provide a basis for evaluating satisfaction of ancillary providers for other NBCCEDP programs as well as other community-based health services. Since this was a pilot study, another limitation is the small sample size. Further study is needed to determine whether the findings in this pilot study are applicable to other states or similar community-based health programs.

Providers of ancillary health services may have lesser expectations for financial gain, especially from community-based health programs intended for the care of low-income and uninsured patients. The results imply that health care programs that do not provide significant financial gain to health care providers must exert efforts to render efficient reimbursement procedures, good relations, and lesser administrative demands.

Factors for satisfaction among ancillary health service providers are rarely included in health care personnel needs and satisfaction studies. Moreover, community-based programs such as the Nevada WHC and other funded programs through the NBCCEDP rely on a small pool of providers for their services. It is necessary to target these factors associated with provider satisfaction in order to retain and improve provider participation and services. More studies are needed to understand the dynamics of job satisfaction among health professionals in community health care settings as these issues have implications for quality improvement in program implementation and planning of health care services.

## ACKNOWLEDGMENTS

This study was funded through a grant from the Centers for Disease Control and Prevention (CDC) (Grant Number U55/CCU922006-02). The authors would like to thank Salome Kapella, MPA, and Graciela Tena de Lara for their assistance in conducting the survey. An additional acknowledgment is made to the staff of the Women's Health Connection, Rocio Flores together with Deborah McBride and Andy DuMond of the Nevada State Health Division.

## BIOSKETCHES

**Emmanuel C. Gorospe, MD, CHCE,** is a consultant for the Center for Ethics and Health Policy and the Nevada Multicultural Coalition for End-of-Life Issues. He was a founding member and codirector for the first university-based hospice and palliative care program in the Philippines. His current research interests include community health services, health behaviors, and end-of-life care. E-mail: GorospeE@unlv.nevada.edu

**Christopher R. Cochran, PhD,** is an associate professor of health care administration and policy at the University of Nevada Las Vegas School of Public Health. He has coauthored several articles, technical reports, and professional papers concerning the health care needs of the medically underserved and the organizations that provide services to such populations. His work has focused on management issues and quality improvement in public organizations. E-mail: Chris.Cochran@unlv.edu

**Elena E. Cabb, BS,** is a postgraduate researcher. At this time, she is completing her master of public health degree at the University of Nevada Las Vegas. She is currently working on projects concerning women and children's health in Nevada. E-mail: CabbE@unlv.nevada.edu
